# The Pelican

**Published:** 1849-04

**Authors:** 


*The Pelican.*—This name was applied by the older dentists to a clumsy and awkwardly contrived instrument, employed in the extraction of teeth; numerous engravings of which are contained in the works of Fauchard,

Bourdet and other writers of the eighteenth century. As the instrument had long since fallen into disuse, we were not a little amused as well as surprised, on being informed a few weeks since by Dr. Hartner of Illinois, that a gentleman, we believe from the south, claimed to be the inventor of it, and had actually obtained a patent, securing to himself the exclusive right of its manufacture, and sale in the United States. On a recent visit to Washington City, Dr. Hartner met with the gentleman, who was offering it for sale, and as a matter of curiosity, purchased one of them and presented to us for the Museum of the Baltimore College of Dental Surgery.

